# *Candida auris*: epidemiological situation, laboratory capacity and preparedness in the European Union and European Economic Area*, January 2018 to May 2019

**DOI:** 10.2807/1560-7917.ES.2020.25.12.2000240

**Published:** 2020-03-26

**Authors:** Diamantis Plachouras, Felix Lötsch, Anke Kohlenberg, Dominique L Monnet, the Candida auris survey collaborative group

**Affiliations:** 1 European Centre for Disease Prevention and Control (ECDC), Stockholm, Sweden; 2 The members of the Candida auris survey collaborative group are listed at the end of the article

**Keywords:** Candida auris, epidemiology, laboratory capacity, preparedness

## Abstract

Between January 2018 and May 2019, 349 cases of *Candida auris* were reported in the European Union/European Economic Area*, 257 (73.6%) colonisations, 84 (24.1%) bloodstream infections, seven (2.0%) other infections and one case of unknown infection/colonisation status (0.3%). Most cases (97.1%, n = 339) were reported from Spain or the United Kingdom, but also for the first time in Greece, the Netherlands and Poland. Laboratory capacity and preparedness has improved since January 2018.

*Candida auris* is an emerging pathogenic fungus that is causing difficult-to-control outbreaks of invasive healthcare-associated infections associated with patient carriage and environmental surface contamination. In 2018, the European Centre for Disease Prevention and Control (ECDC) performed a survey to assess the situation regarding *C. auris* epidemiology, control measures and laboratory capacity for detection in the European Union/European Economic Area (EU/EEA)* [1]. The information from this survey was used to increase awareness about *C. auris* and to assess the need for further action to support *C. auris* detection, prevention and control activities in EU/EEA countries. In order to assess the evolution of the epidemiological situation and the current state of preparedness in the EU/EEA, the ECDC conducted a similar survey in 2019. 

## Survey

In June 2019, we invited the national focal points in EU/EEA countries to complete an online survey. The focal points or their deputies are nominated experts who collaborate with the ECDC on healthcare-associated infections. This survey was an update of the 2018 survey and consisted of 14 questions, four on the country’s epidemiological situation, six on laboratory capacity and four on preparedness and response. A case was defined as a patient in whom *C. auris* was detected irrespective of whether it was an infection or carriage.

## Epidemiological situation

**Figure 1:**
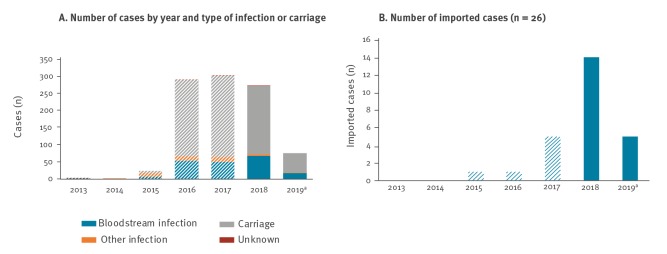
Reported cases of *Candida auris* infection and carriage, EU/EEA, January 2013–May 2019 (n = 969)

^a^ 2019 data until May.

EEA: European Economic Area; EU: European Union.

We received replies from 29 of 30 EU/EEA countries. In total, nine countries reported 349 cases from 1 January 2018 to 31 May 2019. Cases were reported from Spain (n = 291), the United Kingdom (UK) (n = 48), Germany (n = 3), the Netherlands (n = 2), Austria (n = 1), France (n = 1), Greece (n = 1), Norway (n = 1) and Poland (n = 1). Bloodstream infections accounted for 84 (24.1%) cases, other infections for seven (2.0%) cases and carriage for 257 (73.6%) cases (Figure 1). Information on infection or carriage status was missing in one case (0.3%). Fifteen EU/EEA countries reported that they did not detect any case of *C. auris* infection or carriage in the given period. No answer was received from the Czech Republic. Five countries (Latvia, Lithuania, Luxembourg, Portugal and Romania) reported that information on the detection of cases of *C. auris* infection or carriage was not available at the national level.

Dashed bars indicate that data are from the previous survey.

**Figure 2:**
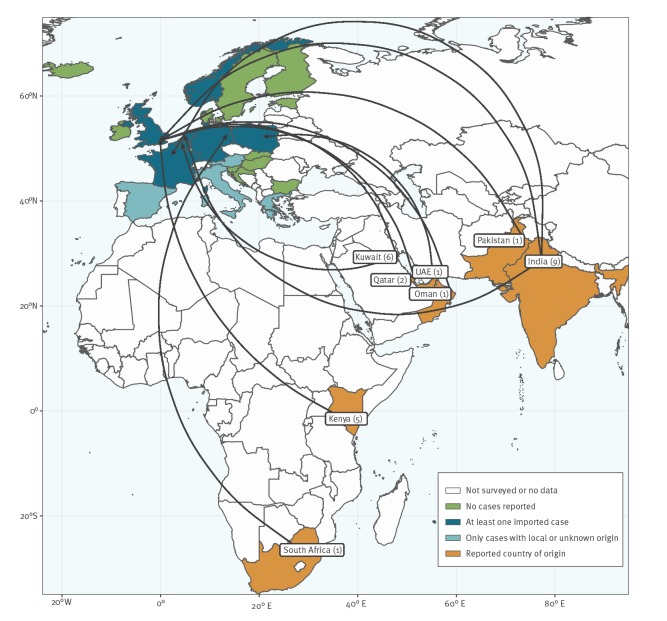
Geographical distribution and reported origin of *Candida auris* infection and carriage, EU/EEA, January 2013–May 2019 (n = 26)

EEA: European Economic Area; EU: European Union.

Among the cases, 324 (92.8%) were locally acquired, 19 (5.4%) were considered imported because they had a history of hospitalisation in a country with reported cases, and for six (1.7%) cases the place of acquisition was unknown. Among the 19 imported cases from January 2018 to May 2019, eight were imported from India, four from Kenya, two from Qatar and one each from Kuwait, Oman, Pakistan, South Africa and the United Arab Emirates. Figure 2 summarises the geographical distribution of *C. auris* cases in EU/EEA countries and the reported origin of imported cases for the period January 2013 to May 2019.

The number of cases reported as imported into the EU/EEA by probable country of origin is indicated in parentheses

Between January 2013 and May 2019, two countries reported local transmission in hospitals: Spain (n = 2 hospitals) and the UK (n =6 hospitals). All these hospitals were large tertiary care centres in large cities. Inter-facility transmission was reported in both Spain and the UK. At the time of the survey in June 2019, there was ongoing transmission in two hospitals in Spain.

## Laboratory capacity

Twenty-two of the 29 responding EU/EEA countries reported the presence of a reference laboratory for *C. auris*. In all but one of the reference laboratories, antifungal susceptibility testing was available (Table). Methods for the identification of *C. auris* were available, including MALDI-TOF in 22 countries, sequencing of D1/D2 locus in eight countries and sequencing of internal transcribed spacer in seven countries. Guidance for laboratory testing was available in 13 countries.

**Table:** National laboratory capacity for *Candida auris* identification and testing and public health measures taken in response to *C. auris*, EU/EEA, January 2018–May 2019 (n = 29)

Country	Notifiable	Mycology reference laboratory / laboratory with reference function	Antifungal susceptibility testing at reference laboratory	Provision of reference testing to hospital laboratories	Guidance for laboratory testing	Guidance for infection control	Prospective surveillance	Retrospective surveillance	Clinical alert in 2018 ^a^	Laboratory alert in 2018 ^a^
Austria	N	Y	Y	Y	Y	Y↑	Y↑	Y	Y	Y
Belgium	N	Y	Y	Y↑	Y↑	Y↑	N	Y↑	N	Y
Bulgaria	N	Y	Y	Y	N	N	N	N	N	Y
Croatia	N	Y	Y	Y↑	N↓	Y	Y↑	N	Y	Y
Cyprus	N	N	N	N	N	N	N	N	N	N
Denmark	N	Y	Y	Y^b^	Y↑	N	Y	Y^b^	N	Y
Estonia	N	N	N	N↓	Y↑	N^c^	N	N	Y	N
Finland	Y	Y	Y	Y↑	Y↑	Y↑	Y↑	N	Y	Y
France	Y	Y	Y	Y	Y↑	Y↑	Y	Y	Y	Y
Germany	N	Y	Y	Y	Y	Y↑	N↓	N	N	Y
Greece	Y	Y	Y	Y	Y↑	Y↑	Y↑	Y	Y	Y
Hungary	N	Y	Y	Y↑	N	N	N	N	N	N
Iceland	N	Y	Y	Y	N	N	N↓	N	N	N
Ireland	Y	N	N	N^d^	N	N	Y	N	Y	N
Italy	Y	N	N	N	N	N	N	N	Y	Y
Latvia	N	N	N	N	N	N	N	N	N	N
Lithuania	N	Y	Y	N	N	N	N	N	N	N
Luxembourg	N	Y	N	Y↑	N	N	N	N	N	N
Malta	N	Y	Y	Y↑	N↓	N	N	N	N	Y
Netherlands	N	Y	Y	N	Y↑	N	N	Y	Y	Y
Norway	N	Y	Y	Y↑	Y	N↓	N	N	N	Y
Poland	N	N	N	N	N	N	N	N	N	N
Portugal	N	Y	Y	Y↑	Y↑	Y↑	Y↑	Y↑	N	Y
Romania	N	N	N	N	N	N	N	N	N	N
Slovakia	N	Y↑	Y↑	N	N	N	N	N	N	N
Slovenia	N	Y	Y	Y	Y↑	Y↑	Y	N	N	Y
Spain	N	Y	Y	Y	N	Y↑	N	N	Y	Y
Sweden	N	Y	Y	Y	N	N	N	N	Y	Y
United Kingdom	N	Y	Y	Y	Y	Y	Y	Y	N	N

EEA: European Economic Area; EU: European Union; N: no; Y: yes.

^a^ Only refers to 2018 and does not include alerts issued in previous years.

^b^ Since 2010, all fungal bloodstream isolates in Denmark have been sent to the reference laboratory as part of the national fungaemia surveillance programme. From 2004 to 2009, only two thirds of the country was covered by this surveillance programme.

^c^ *Candida auris* is addressed in general infection control guidelines.

^d^ Advice for samples to be sent to the United Kingdom for reference testing.

Changes as compared with the previous survey that assessed the situation as at January 2018 are marked with ↑ (no to yes) or ↓ (yes to no).

## Public health preparedness and response

At the time of the survey, infection with or carriage of *C. auris* was notifiable in only five EU/EEA countries (Finland, France, Greece, Ireland and Italy) (Table), but 11 other countries (Croatia, Cyprus, Estonia, Hungary, Lithuania, Luxembourg, Norway, Portugal, Romania, Slovakia and Slovenia) were considering making *C. auris* a notifiable disease. Guidance for the clinical management of *C. auris* as well as guidance for infection control was available in seven countries and 11 countries, respectively. In six countries, retrospective surveillance was ongoing, whereas prospective surveillance was performed in 10 countries. In 2018, clinical and laboratory alerts were issued in 11 and 17 countries, respectively.

## Discussion

*Candida auris* is an emerging *Candida* species causing bloodstream and other infections, mostly in severely ill patients with serious underlying medical conditions. It has been detected on five continents within a few years of its first identification in 2009. Identification of *C. auris* requires specialised laboratory methodology such as matrix-assisted laser desorption/ionisation-time of flight (MALDI-TOF) mass spectrometry or sequencing of specific gene loci [2,3]. Reliance on traditional methods may lead to misidentification. *Candida auris* is usually resistant to fluconazole [4] and has been associated with resistance to multiple antifungal drug classes including echinocandins and amphotericin B [5]. The case fatality of bloodstream infections with *C. auris* has been reported to be up to 60% and the infections may be difficult to treat because of resistance to multiple antifungal agents [2,6]. *Candida auris* is able to colonise surfaces and survive outside the host [7] and unlike other *Candida* species, *C. auris* has been linked to outbreaks in healthcare settings. These characteristics render *C. auris* a potential threat for European healthcare facilities. 

The number of new cases in 2018 in the EU/EEA (n = 273) was comparable to those in 2016 and 2017 (n = 290 and 303, respectively) [1]. However, three countries (Greece, the Netherlands and Poland) reported cases for the first time in 2018. After completion of this survey, one case of *C. auris* infection was reported in Italy in September 2019 [8], thus increasing the number of EU/EEA countries that have so far detected cases to 10.

In European countries outside the EU/EEA, one case of colonisation with previous hospitalisation in Spain was reported in Switzerland in 2017 [9] and a hospital outbreak affecting 49 critically ill patients was reported in Russia in 2016 and 2017 [10]. In the EU/EEA, hospital outbreaks have occurred in Spain and the UK, as already reported in the 2018 survey [1], with spread between hospitals documented in both countries. All other countries that identified cases of *C. auris* only reported sporadic cases and did not detect further local transmission. However, information on the investigation of contacts of the sporadic cases is not available and undetected transmission cannot be excluded. Data from the UK show that early detection of outbreaks in combination with isolation, enhanced infection control measures and screening can halt *C. auris* outbreaks [11]. The repeated occurrence of sporadic cases and the detection of cases in countries that were not previously affected underlines the continuous risk of introduction of *C. auris* into hospitals and other healthcare institutions in EU/EEA countries. Five per cent of all reported cases in this survey were classified as imported. Almost half of these cases had previously been hospitalised in India. German authors reported on a case series of seven patients with *C. auris* infection or colonisation. Among these, six had previously been hospitalised in another country: one each in Afghanistan, Dubai, Oman, Russia, Saudi Arabia and the sixth patient was hospitalised in several other countries (Kenya, the UK and the United States) in addition to Germany [12]. This highlights the relevance of screening patients with previous hospitalisation in high prevalence regions for carriage of *C. auris* in addition to multidrug-resistant bacteria. However, a number of cases reported in this survey did not have a history of previous travel or hospitalisation abroad nor were linked to known clusters. The identification of such cases may be an indication of undetected dissemination and is a cause for concern. Furthermore, international communication is crucial, using, if necessary, the European Early Warning and Response System or the International Health Regulations, in cases where *C. auris* has not been reported from the source institution/country.

Species identification of *C. auris* can be challenging but is a prerequisite for adequate treatment and effective infection control measures [2,3,13]. Yet, seven EU/EEA countries still reported that a reference laboratory did not exist in their country. Thus, cases of *C. auris* may still be misidentified, with the potential to spread in healthcare settings. A survey in April 2018 in Belgium and Luxembourg, only 57.7% of 142 laboratories were able to correctly identify a strain of *C. auris* [14], whereas in Germany, 85% of 233 participating laboratories correctly identified *C. auris*. Moreover, six participating countries reported, both in the 2018 and the 2019 surveys, not having issued clinical or laboratory alerts on *C. auris*. On the other hand, provisions are now in place and reference testing is being offered to hospital laboratories in 18 countries compared with 12 in the 2018 survey. Progress has also been made in developing and introducing guidelines. Whereas in the 2018 survey only six countries reported guidance on laboratory testing [1], 13 did so in 2019. Guidance for clinical management is now available in seven EU/EEA countries compared with three in the 2018 survey, and guidance for infection control is now reported from 11 countries in contrast to only three in the 2018 survey. In addition, the number of countries that performed retrospective surveillance at the time of the 2019 survey had increased from six to 10, whereas only six countries conducted prospective surveillance. Ensuring laboratory capacity and increasing awareness is addressed in the ECDC rapid risk assessment [6].

## Conclusion

Both introduction of *C. auris* from countries outside of the EU/EEA and spread of *C. auris* within EU/EEA countries are ongoing. Although overall laboratory capacities and public health preparedness and response to *C. auris* are improving, not all EU/EEA countries are equally well prepared. A collective commitment in all EU/EEA countries to improve awareness and optimise laboratory capacities, surveillance and infection control is crucial to prevent further spread of *C. auris* in the EU/EEA.

## *Note

At the time of writing this article, the United Kingdom was part of the European Union.

## Candida auris survey collaborative group

Austria: Cornelia Lass-Flörl, Robert Muchl, Birgit Willinger; Belgium: Katrien Lagrou; Bulgaria: Ivva Philipova; Croatia: Ana Budimir; Cyprus: Linos Hadjihannas; Denmark: Maiken Cavling Arendrup; Estonia: Pille Märtin; Finland: Outi Lyytikäinen, Jari Jalava; France: Françoise Dromer; Germany: Oliver Kurzai; Greece: Georgia Vrioni, Flora Kontopidou; Hungary: Andrea Kurcz, Judit Zala; Iceland: Gudrun Sigmundsdottir; Ireland: Karen Burns; Italy: Maria Luisa Moro; Latvia: Kate Vulāne; Lithuania: Rolanda Valintėlienė; Luxembourg: Monique Perrin; Malta: Elizabeth Anne Scicluna; the Netherlands: Sabine de Greeff; Norway: Oliver Kacelnik; Poland: Aleksander Deptuła; Portugal: Maria do Rosário Rodrigues; Romania: Roxana Serban; Slovakia: Mária Štefkovičová; Slovenia: Mojca Serdt; Spain: Ana Alastruey-Izquierdo, Pilar Gallego Berciano; Sweden: Sara Byfors, UK: Colin Brown.
